# Mutation Changes in the preC/Core Promoter in HBeAg-Positive Patients With Chronic Hepatitis B During Interferon Therapy

**DOI:** 10.1097/MD.0000000000002657

**Published:** 2016-02-08

**Authors:** Yan Geng, Xiangling Wang, Xiaolan Lu, Xiaokang Wu, Nan Xu, Lei Han, Jiru Xu

**Affiliations:** From the Department of Laboratory (YG, XW, XW, NX); Department of Digestive, The Second Affiliated Hospital (XL); and Department of Immunology and Pathogenic Biology, Health Science Center, Xi’an Jiaotong University, Xi’an, People's Republic of China (LH, JX).

## Abstract

To study the changes in 3 mutations related with hepatitis B e antigen (HBeAg) in patients with HBeAg-positive chronic hepatitis B (CHB) during interferon therapy.

HBeAg seroconversion is a major therapeutic milestone for patients with HBeAg-positive CHB. The precore mutation G1896A and the basal core promoter mutations A1762T/G1764A are 3 important mutations that affect the expression of HBeAg; however, the change of these 3 mutations in CHB patients during interferon therapy has not yet been evaluated.

Sixty-four treatment-naive patients with HBeAg-positive CHB were treated with interferon for 48 weeks and followed up for 24 weeks. Serum samples were collected from all of the participants at different time points and then subjected to viral DNA extraction. The precore and basal core promoter sequences were determined using nested PCR and direct sequencing. The treatment outcomes were confirmed both at the end of therapy and the follow-up period, and the results were compared between patients with mutant and wild-type virus.

No significant difference in HBeAg loss and HBeAg seroconversion was observed between patients with mutant versus wild-type virus although the portion of patients who achieved HBeAg loss/seroconversion with mutant virus was a little higher than in patients with wild-type virus. Once a mutation exists, it is not replaced with the wild-type sequence during interferon therapy and follow-up; moreover, our results show that mutants stably coexist with the wild-type virus during interferon therapy.

This study shows the changes in 3 mutations affecting the expression of HBeAg during interferon therapy. However, additional studies with a larger sample size and more sensitive detection methods are needed to uncover the underlying mechanism and clinical significance of these results.

## INTRODUCTION

Hepatitis B virus (HBV) infection is a major cause of chronic liver disease, which may progress to liver cirrhosis, liver failure, and hepatocellular carcinoma.^1^ An estimated one-third of the world's population has been infected with HBV, with approximately 240 million chronic hepatitis B (CHB) patients worldwide, including 93 million in China^2,3^ (http://www.who.int/mediacentre/factsheets/fs204/en/, WHO Fact sheet N 204, updated July 2015). Antiviral therapy for CHB can significantly reduce the progression of HBV infection.^4–6^ At present, 2 classes of agents, including peginterferon (Peg-IFN), which stimulates the immune response to HBV, and nucleot(s)ide analogs (NAs), which directly suppress viral replication, are recommended as first-line therapies according to international guidelines for CHB treatment.^7–10^ Hepatitis B e antigen (HBeAg) seroconversion from HBeAg-positive to anti-HBeAg-positive represents a major therapeutic milestone for patients with HBeAg-positive CHB.^11^

Due to a lack of proofreading and the spontaneous error rate of viral reverse transcriptase, the HBV genome evolves and multiple variants appear during long-term replication under the antiviral pressure of host immunity or specific therapy.^12,13^ Of all the HBV variants, precore and basal core promoter (BCP) mutations are commonly shown to affect the expression of HBeAg.^14–16^ The most common precore variant contains a G to A substitution at nucleotide 1896 (G1896A), which prevents the production of HBeAg by introducing a stop codon in the precore region.^17^ The most frequent BCP mutation is a double mutation involving an A to T substitution at nucleotide 1762 and a G to A substitution at nucleotide 1764 (A1762T/G1764A), which suppress the expression of HBeAg but enhance viral genome replication in vitro.^13,18,19^ Because both of these BCP mutants are associated with decreased HBeAg levels and disease progression,^15,16^ their evolution may also reflect an evasion of immune mechanisms targeting HBeAg and thus may be associated with selection during HBeAg seroconversion.^20,21^

The evolution of these 2 mutants has been studied in patients with CHB who either experienced HBeAg seroconversion naturally^13,21^ or through NA treatment^22,23^; however, the changes in A1762/G1764 and G1896 have not been investigated in patients with CHB during Peg-IFN treatment. Moreover, Peg-IFN functions as both an immunomodulatory agent and an antiviral agent, which is different from the single antiviral effect of NAs. Thus, the present study aimed to study the changes in the A1762/G1764 and G1896 mutations in treatment-naive patients with HBeAg-positive CHB during Peg-IFN treatment.

## MATERIALS AND METHODS

### Subjects

This study was conducted in accordance with the guidelines of the Declaration of Helsinki, and the protocol was approved by the Ethics Committee of the Second Affiliated Hospital of Xi’an Jiaotong University. Written informed consent was obtained from each participant.

Sixty-four treatment-naive patients with HBeAg-positive CHB, who were hospitalized and followed in the Department of Digestive, the Second Affiliated Hospital of Xi’an Jiaotong University from September 2011 to December 2012, were enrolled in this study. Patients with CHB were diagnosed according to the Asian-Pacific Guideline.^24^ The patient was included if he or she: was hepatitis B surface antigen (HBsAg) positive for at least 6 months before enrolment; was HBeAg positive at the time of screening; had elevated serum alanine aminotransferase (ALT) levels (2–10 times the upper limit of normal); had HBV DNA ≥10^4^ copies per milliliter; received no previous antiviral therapy. The patient was excluded if he or she was coinfected with other hepatitis viruses or human immunodeficiency virus; or had other liver diseases.

All of the enrolled 64 patients received Peg-IFN (Roche, Basel, Switzerland) treatment for 48 weeks. After treatment, the patients were followed up for 24 weeks. At the indicated time points (0, 12, 24, 48, and 72 weeks), fasting serum was collected and stored at −80 °C until subsequent analysis.

### Laboratory Measurements

Serum ALT and virological markers were measured at the Clinical Laboratory of our hospital. The serum ALT levels were detected by an automatic biochemical machine (Olympus, AU2700, Tokyo, Japan). Serum HBsAg, HBeAg, and anti-HBe levels were tested using commercial assays with Abbott Architect I2000 (Abbott Laboratories, Abbott Park, IL). Hepatitis B virus DNA was quantified using a real-time quantitative PCR assay (Kehua, Shanghai, China) with a lower detection limit of 500 copies/mL with an ABI-7000 machine (Roche, Basel, Switzerland).

### Viral DNA Extraction, Polymerase Chain Reaction Amplification and Sequencing

The precore and BCP sequences were determined using nested PCR and direct sequencing. Hepatitis B virus DNA was extracted from each serum sample using the QIAamp DNA blood mini kit (Qiagen, Hilden, Germany) following the manufacturer's instructions. The extracted DNA was used as a template for the amplification of the precore and BCP regions as well as S gene. The HBV gene fragment (nt 1653–1974) containing the precore and BCP regions and fragment (nt 46–1022) containing S gene were amplified via nested PCR before sequencing.

For fragment containing the precore and BCP regions, nested PCR was performed using the outer primers BCP1-F-5′ TCGCATGGAGACCACCGTGA 3′ and BCP1-R-5′ ATAGCTTGCCTGAGTGC 3′, and the inner primers BCP2-F-5′ CATAAGAGGACTCTTGGACT 3′ and BCP2-R-5′ GGAAAGAAGTCAGAAGGC 3′. The first round of PCR was performed as follows: first denaturation at 94 °C for 5 minutes, followed by 30 cycles of 94 °C for 1 minutes, 55 °C for 30 seconds and 72 °C for 1 minutes, and last extension at 72 °C for 10 minutes. Using the first-round PCR products as templates, a second-round PCR amplification was performed as follows: first denaturation at 94 °C for 5 minutes, followed by 30 cycles of 94 °C for 45 seconds, 52 °C for 30 s and 72 °C for 1 minutes, and last extension at 72 °C for 10 minutes. The PCR products were purified using a commercial pure kit (Omega, Norcross, GA) and then submitted for direct sequencing (Sangon, Shanghai, China).

For fragment containing S gene, PCR assay was performed as above described with outer primers 5′ CCTCCACCAATCGGCAGTCAG3′ and 5′-GCAGCAAAGCCCAAAAGACCC-3′, and inner primers 5′-CCTGTATTTTCCTGCTGGTGGCTCC-3′ and 5′-GCAGCAAAGCCCAAAAGACCC-3′. Hepatitis B virus genotype assignment was based on phylogenetic analysis of the fragment for S gene sequencing as previously reported.^25^

### Statistical Analysis

Statistical analyses were performed using SPSS 19.0 statistics software (SPSS, Chicago, IL). Data are presented as the mean and standard deviation (mean ± SD). Hepatitis B surface antigen, HBeAg, and HBV DNA data were transformed into log scale. Continuous variables were compared using *t* tests. The percentage of HBeAg loss or HBeAg seroconversion between the mutant and wild-type virus was compared using a χ^2^ test. Two-sided *P*-values <0.05 were considered statistically significant.

## RESULTS

### Demographic and Clinical Characteristics

The baseline characteristics of the studied patients are shown in Table 1. The mean age was 30 ± 8 years (age range: 21–55 years). Of the 64 patients, 31 patients showed wild-type virus, 12 (18.8%) patients showed the precore G1896A mutation (all of the 12 patients coexist wild-type 1896G, and 2 of them coexist A1762T and G1764A BCP mutations), 20 (31.3%) patients showed the BCP A1762T mutation (18 of 20 simultaneously presented the G1764A mutation), and 21 (32.8%) patients showed the G1764A mutation. No significant difference was observed in age, sex, serum ALT level, or serum HBsAg level between patients with mutant virus and wild-type virus. The number of HBV DNA copies was significantly lower in patients with mutant virus than in patients with wild-type virus (*P* < 0.001). In addition, the serum HBeAg level was significantly lower in patients with the precore G1896A mutant compared with patients with BCP mutant or wild-type virus (*P* < 0.001). No significant difference in HBV genotype was observed between patients with mutant virus and wild-type virus, except that the portion of genotype B was a little higher in patients with wild-type virus.

**TABLE 1:**
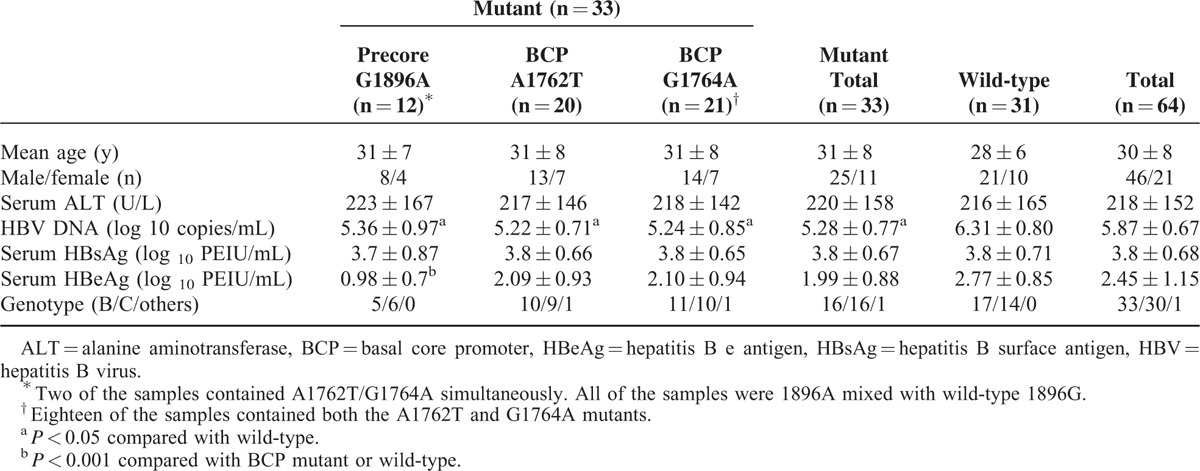
Demographic, Clinical, and Serum Characteristics of Patients With Chronic HBV Infection

### Treatment Outcomes at the End of Treatment and Follow-up

All of the patients received Peg-IFN-α treatment for 48 weeks and were followed up for 24 weeks. At the end of treatment, 42.2% (27/64) of the patients achieved HBeAg loss, 39.1% (25/64) of the patients achieved HBeAg seroconversion, 9.4% (6/64) of the patients achieved HBsAg loss, and 6.3% (4/64) of the patients achieved HBsAg seroconversion; however, more patients achieved HBeAg seroconversion than HBsAg seroconversion at the end of the follow-up period. In addition, 48.4% (31/64) of the patients achieved HBeAg loss and 43.8% (28/64) of the patients achieved HBeAg seroconversion at the end of the follow-up period. Only 4.7% (3/64) of the patients achieved HBsAg loss, and 1 patient achieved HBsAg seroconversion at the end of the follow-up period (Table 2).

**TABLE 2:**
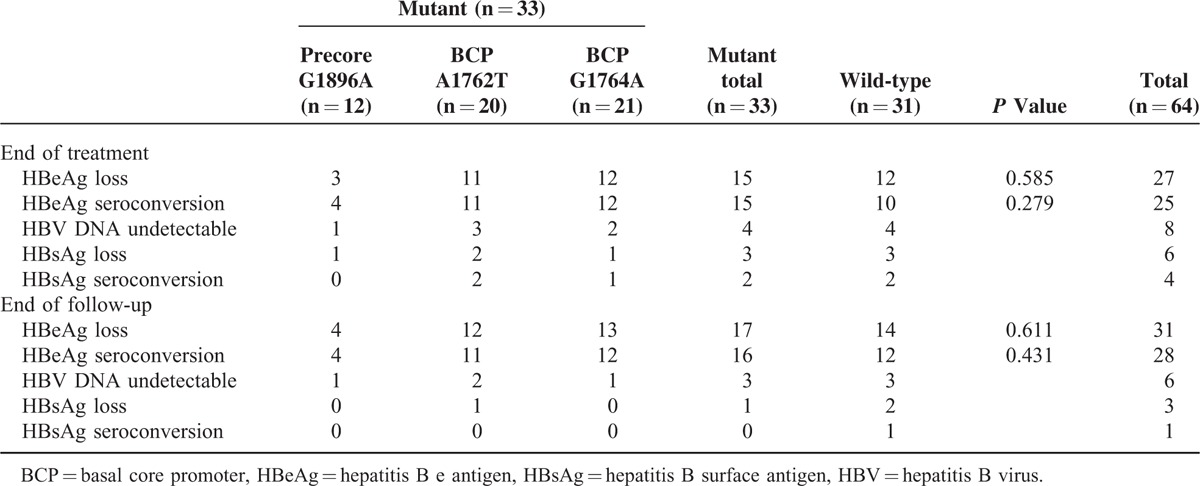
Treatment Outcomes and Follow-up Results

Of the patients with viral mutants, 51.5% (17/33) achieved HBeAg loss, and 48.5% (16/33) of the patients with wild-type virus achieved HBeAg seroconversion. In comparison, among patients with wild-type virus, 45.2% (14/31) showed HBeAg loss and 38.7% (12/31) showed HBeAg seroconversion. Although the portion of patients achieved HBeAg loss/seroconversion in the mutant group was a little higher than that in the wild-type group, no significant difference was found in HBeAg loss or HBeAg seroconversion between patients with mutant or wild-type virus (*P* > 0.05).

### Dynamic Changes of the Wild-type Virus During Treatment and Follow-up

Of the 31 patients with wild-type virus, 2 patients harbored virus with both the BCP A1762T and G1764A mutations and 1 patient harbored virus with the precore G1896A mutation at the 12-week time point. There were 4, 3, and 2 patients with the A1762T, G1764A, and G1896A mutations at week 24 respectively; there were 7, 7, and 3 patients with the A1762T, G1764A, and G1896A mutations at week 48 respectively; and there were 9, 9, and 6 patients with the A1762T, G1764A, and G1896A mutations at week 72, respectively (Table 3). All of the patients with mutant virus were also infected with wild-type virus. At the end of the follow-up period, only 2 patients harbored a virus with 3 mutations, and 3 patients showed undetectable HBV DNA levels.

**TABLE 3:**
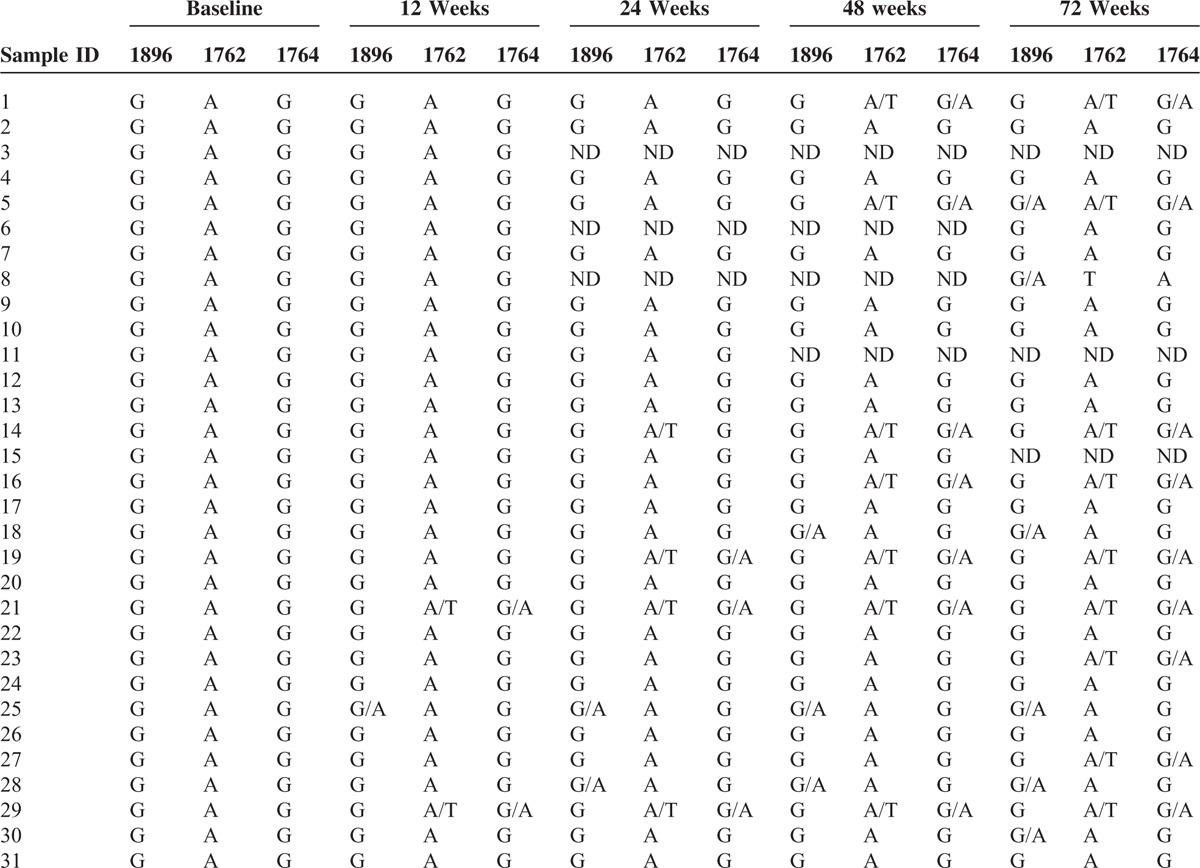
Changes in the Wild-type Virus During Treatment and Follow-up

### Dynamic Changes in the Basal Core Promoter A1762T and G1764A Mutants during Treatment and Follow-up

Of the 23 patients with the BCP A1762T or G1764A mutant, none showed reversion to the wild-type sequences (Table 4). At the end of the follow-up period, in addition to the 2 patients who harbored HBV with 3 mutations at baseline, another 3 patients harbored virus with the precore G1896A mutant, resulting in a total of 5 patients who harbored a virus with 3 mutations. In addition, 2 patients showed undetectable HBV DNA levels.

**TABLE 4:**
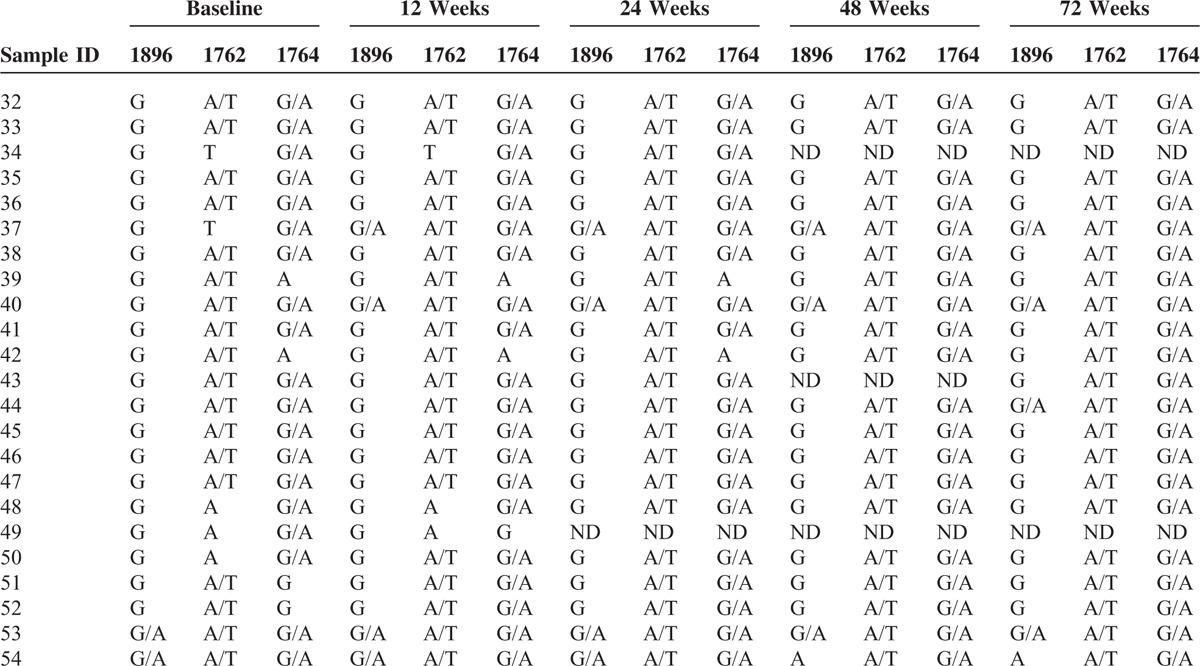
Changes in the Basal Core Promoter A1762T/G1764A Mutants During Treatment and Follow-up

### Dynamic Changes in the Precore G1896A Mutant During Treatment and Follow-up

The changes in the precore G1896A mutant were similar to those observed for the BCP A1762T and G1764A mutants, such that was once the virus had mutated, there was no evidence of reversion to the wild-type sequence (Table 5). At the end of the follow-up period, in addition to the 2 patients who harbored virus with 3 mutations at baseline, another 2 patients harbored virus with BCP A1762T and G1764A mutants, resulting in a total of 4 patients who harbored a virus with 3 mutations. In addition, 1 patient had undetectable HBV DNA.

**TABLE 5:**
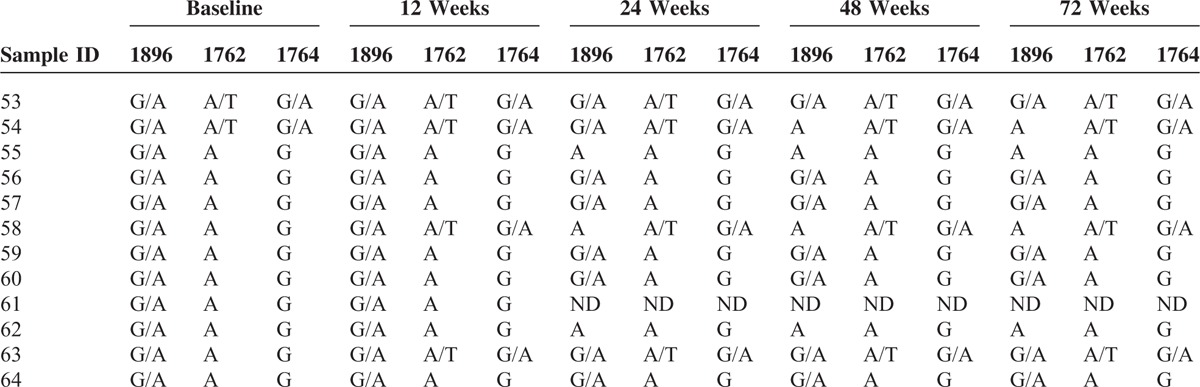
Changes in the Precore G1896A Mutant During Treatment and Follow-up

## DISCUSSION

In the present study, the changes of the BCP A1762T and G1764A mutants and the precore G1896A mutant were analyzed in HBeAg-positive patients with CHB during interferon therapy and follow-up. The data showed that once the virus had mutated at these positions, it did not revert back to the wild-type sequence during interferon therapy and follow-up. Moreover, the majority of the patients with mutants also showed evidence of infection with wild-type virus.

Before therapy, the percentages of the BCP A1762T and G1764A mutants and the precore G1896A mutant were 31.3%, 32.8%, and 18.8%, respectively, and the total mutation percentage was 51.6%, which is comparable to previous reports.^26,27^ At the end of follow-up, almost half of the patients achieved HBeAg seroconversion, and the total mutation percentage of these 3 mutants was 75%, which is consistent with a previous report showing that BCP and precore mutants are more prevalent in HBeAg-negative patients than in HBeAg-positive patients.^28^ Because of the different prevalence of these mutations between HBeAg-negative and HBeAg-positive patients, some researchers have hypothesized that these mutants might be associated with HBeAg seroconversion and 2 studies conducted in Taiwan have confirmed this hypothesis.^29,30^ However, one multicenter study conducted in Europe and Canada reported that these mutations impaired the response to PEG-IFN therapy in HBeAg-positive patients.^31^ In the present study, no significant difference was found in HBeAg seroconversion between the mutants and wild-type virus. However, the portion of patients achieved HBeAg loss/seroconversion in the mutant group was a little higher than that in the wild-type group. The patients in our study, studies conducted by Nie et al^29^ and Yang et al^30^ were all Chinese and the HBV genotypes were mainly B and C, while in the multicenter study conducted by Sonneveld et al^31^ were European and Canadian and the HBV genotypes were mainly A and B. Moreover, Nie et al^29^ observed the natural course of chronic HBV infection and the patients did not received treatment. Yang et al^30^ applied 4 therapy regimens and the majority of the patients received normal IFN or PEG-INF for 24 weeks. Sonneveld et al^31^ treated patients with PEG-IFN alpha-2b for 52 weeks. Thus race, HBV genotype and therapy regimen may contribute to the difference between these studies, and the 3 mutations may not be related with the outcomes of IFN therapy.

Previous studies have shown that lower baseline DNA levels and HBV genotype B contribute a good clinical outcome during IFN therapy.^32,33^ We compared baseline DNA levels and HBV genotype between 2 groups of patients. We found that although the portion of patients infected with HBV genotype B in wild-type group was a little higher than that in mutant group, no significant difference in genotype was observed between mutant group and wild-type group. As for baseline DNA levels, it were significantly lower in patients with mutant virus than that in patients with wild-type virus. All these data indicated that the difference of the portion of patients achieved HBeAg loss/seroconversion between 2 groups might also be related with the baseline DNA levels.

In the present study, we found that these 3 BCP and precore sites, once mutated, did not revert back to the wild-type sequence. This finding is consistent with previous reports with IFN therapy,^29,30,31^ whereas it is not consistent with previous reports with NA therapy.^22,23^ Cho et al^23^ reported that precore mutants did revert back to the wild-type virus in all patients infected with the precore mutant; in addition, the BCP mutants were also replaced by wild-type virus in 75% of patients after 12 months of lamivudine therapy. The study by Suzuki et al^22^ presented a similar result, showing that the precore/BCP mutants could be replaced by the wild-type virus during lamivudine therapy, although the rates of change from the mutants to wild-type virus were much lower than those in the previous study. The difference between our study and these previous reports may be related to the different treatments administered to patients. Lamivudine is well known to specifically inhibit viral replication, and it is therefore common for patients to show undetectable HBV DNA levels during therapy. Specifically, the percentage of patients with undetectable DNA was 50% after 3 years of lamivudine therapy,^22^ which is much higher than that observed in the present study. In addition, the wild-type precore sequence is more associated with lamivudine-resistant mutants during lamivudine therapy.^34^ These results suggested that no therapy bias to mutants and wild-type virus existed in IFN treatment, whereas it may exist in NAs treatment.

We also found that the majority of patients with mutants also harbored wild-type virus, which is in accordance with a previous report studying the mutant changes during the natural course of HBV infection.^29^ Using a sensitive mutation detection method, these authors investigated the quantitative dynamics of BCP and precore mutants before and after HBeAg seroconversion and showed that the mutant percentage displayed a steady increase from <10% to 50% to 100% during the natural course of infection, approximately 3 years before HBeAg seroconversion. After HBeAg seroconversion, the mutant percentage remained high or decreased significantly, but remained above 20%. Because the direct sequencing method we used in the present study could only detect mutations representing at least 20% of the whole HBV population,^35^ both the wild-type virus and mutants were detected in the present study.

Several limitations existed in the present study. First, data from a larger population are needed to confirm our conclusions. Second, direct sequencing is not a highly sensitive method for determining the mutational changes, and it can only detect mutations representing at least 20% of the whole HBV population.^35^ So our results should be interpreted with caution and a more sensitive method of detection is needed to assess viral genetic changes during interferon therapy and understand the corresponding mechanism. Third, a longer follow-up period is needed to further study the dynamic changes in HBV infection following treatment.

In conclusion, we studied the changes in the BCP/precore mutants in treatment-naive patients with HBeAg-positive CHB during Peg-IFN treatment and found that these mutants stably coexist with wild-type virus during therapy. However, further studies are needed to confirm the present results and uncover the underlying clinical significance of the mutation changes.
